# A Novelty in Treatment

**Published:** 1892-03

**Authors:** 


### Editorial.

### A NOVELTY IN TREATMENT.

From the period when Maynard and his co-laborers endeavored to impress the active minds of the profession with the importance of properly preparing and filling pulp-canals, has this apparently simple subject remained in an unsettled state, and is still one of continual controversy in professional gatherings. For more than thirty years after the use of gold was recommended, the number was comparatively few who were willing to antagonize the practice of packing this metal solidly in canals. Gradually other and less complex methods were introduced, at first timidly, but, as time advanced, with boldness and a spirit of aggressiveness, indicating that the use of gold in pulp-canals was gradually passing into disuse. The introduction of oxychloride of zinc, with a better knowledge of how to handle gutta-percha, completed the destruction of old ideas in this direction, and the royal metal, for this purpose, was relegated to oblivion.

Then came a period of mixed treatment. The operation ceased to be confined to one material, and we were presented with cotton,—cotton saturated with carbolic acid or cosmoline; cotton with oxychloride, or the latter material pumped into the canal without any vehicle to carry it; wood from the pine or cedar; metal wires saturated with chloro-percha or without; aristol and antiseptics; the operator who believed in coagulation and the one who denied its possibilities,—indeed, the list has become extended so as to include all the whims and notions that naturally arise in a daily active practice.

It is not surprising that, with this discordant condition of affairs, the subject has come to be regarded at our annual conventions as one scarcely to be tolerated.

Recognizing this, we would not occupy space with its discussion if we did not feel that the last word on this very important topic has not yet been written.

From what has been gathered from the somewhat incongruous *mélange* of practice, it must be evident either that the filling of the pulp-canals is not the important matter it has been supposed to be, or that there is a wide-spread lack of that scientific pathological knowledge so absolutely essential in all treatment.

It is presumed all intelligent operators in dentistry will agree that, with our present knowledge of the germ theory, the important thing to do is to first render the canal aseptic. The second must be to remove all the evidences of putrefaction, and the last to fill it so that it shall absolutely be impervious to fluids both from within and without. This much conceded, that filling is the best that will accomplish this, and that filling is the worst possible which permits absorption of fluids, whether this be imperfectly-packed gold or porous wood.

We have been led to these reflections not from any apparent necessity for an article on this subject, but from the evidence recently presented that from the use of gutta-percha we have been invited to consider the possibility, nay, more, the absolute necessity, of filling all the parts adjacent to the apical foramen, carrying the operation through to the external opening of the fistula produced by alveolar abscess.

This entirely new departure is presented in a paper in this number, prepared by Dr. Eddy and read at a recent meeting of the Academy of Dental Science, Boston. It may not seem strange in these days of startling therapeusis that such a mode of treatment should be suggested, but it does appear remarkable that in this body, composed of the ablest thinkers in the profession in New England, that only one should rise and doubt its propriety. The quotation will explain the position of the essayist.

After using peroxide of hydrogen, and the "whole tract being left in an aseptic condition, . . . gutta-percha is forced . . . through the fistulous opening, closing the canal, as before, with gutta-percha. . . . The old sac at the apex of the root is distended and filled with the solution of gutta-percha, and this remains encysted. Some inflammation often results from forcing the solution of gutta-percha through the fistulous tract, but in a day or two, at most, it usually subsides."

We are not disposed to dogmatically assume that a process antagonistic to received ideas may not have within it the germs of something good; indeed, in these days of heroic surgery such presumption might lead to serious after-reflections on the limitations of human perception. It is, therefore, with no spirit of direct antagonism that we feel impelled to call attention to this remarkable latter-day treatment of pericemental inflammation and the resulting fistula, but rather to broaden, if practicable, thoughts upon this subject.

Perhaps it is not possible to intelligently consider this matter at all unless an outline be made of the condition of a tooth after the destructive effects of alveolar abscess. The focus of inflammation may be supposed to be at the apex of the root. This means primarily congestion of the blood-vessels of the pericementum, the infiltration of the fibrous tissue with the corpuscles and fluid contents of the blood, and final thickening of the membrane and its subsequent separation from the cement and the formation of the, so-called, sac. This is effected by the pressure of the fluid elements between the wall of the sac and the tooth. This means that the cement, and consequently the tooth, to the extent of separation, has lost its means of nutrition. The final result is necrosis of that portion. After an indefinite period a fistula is established and the confined pus escapes. These pathological truisms need only to be stated to intelligent readers. If, then, this condition exists in all cases of abscess we are forced to the conclusion that no tooth having been once placed in this abnormal pathological state can ever again regain, while the dead portion remains, a condition of absolute health.

Having arrived at this point the essayist proposes to fill the canal with a solution of gutta-percha and chloroform, and pump sufficient through the foramen to distend the sac and fill the fistula, and then the whole is left to become "encysted."

It is a recognized fact that nature tolerates with wonderful kindness foreign bodies, but we have yet to learn that a tissue in a pathological state will accept the intrusion of a foreign element and be restored through its influence to a state of health and a reparation of parts. Yet this is exactly what must be done to make the plan proposed a success.

In considering this subject, the safe course may be adopted of endeavoring to arrive at correct conclusions through facts presented in clinical experience.

Dentists have been taught that the forcing of foreign matter through the apical foramen means, sooner or later, an increase of the local inflammation and its possible extension to surrounding parts. They have recognized the truth of this whenever they have been unfortunate in forcing any substance through this natural opening or one artificially made. There has been no exception to this. Gutta-percha has been equally as bad as cotton, and portions of gold equally as injurious as a wire penetrating the foramen. It may be argued with some force that this is not analogous to a mass injected until all parts are thoroughly filled, but to our conception the greater includes the less, and means, if it means anything, only an increase of the irritation and subsequent destruction of parts.

The experience of the writer is, probably, in accord with the majority, that the presence of a foreign body at that part of the human organism simply invites an increase of inflammation.

Whether it has been from an extension in the use of agents, in a semi-fluid state, in canals, or through carelessness in pressing material beyond the prescribed limits, it is certainly true that the number of cases of necrosis of the alveolus is on the increase. This may have its origin in the necrotic condition of the cement surrounding the root at the apex, but in several recent cases it has been traced directly to the constant presence of gutta-percha. In one case of serious character involving extensive destruction of adjacent tissues, this was so clearly apparent that we have been forced to the conclusion that he who attempts the operation proposed by the essayist must be pronounced guilty of experimenting on his patients without proper warrant.

The essayist fails to give any philosophical reasons for the faith he maintains, nor do those who so fully endorse the practice. That they have had apparent success must be conceded, but that does not mean that the process is theoretically correct, or that it has been scientifically demonstrated.

As the matter stands we cannot encourage any radical departure from established methods, but would rather advise the most conservative handling of the pulp-canals, if the operator wishes to avoid mortification and loss in attempting those things not in accord with recognized pathological experience and treatment.

